# Development of Novel Anti-Leishmanials: The Case for Structure-Based Approaches

**DOI:** 10.3390/pathogens11080950

**Published:** 2022-08-22

**Authors:** Mohini Soni, J. Venkatesh Pratap

**Affiliations:** 1 Biochemistry and Structural Biology Division, CSIR-Central Drug Research Institute, Sector-10, Jankipuram Extension, Sitapur Road, Lucknow 226031, India; 2 Academy of Scientific and Innovative Research (AcSIR), Ghaziabad 201002, India

**Keywords:** leishmaniasis, neglected tropical disease, purine salvage pathway, folate biosynthesis pathway, peroxisomal pathway, structure-based drug design

## Abstract

The neglected tropical disease (NTD) leishmaniasis is the collective name given to a diverse group of illnesses caused by ~20 species belonging to the genus *Leishmania*, a majority of which are vector borne and associated with complex life cycles that cause immense health, social, and economic burdens locally, but individually are not a major global health priority. Therapeutic approaches against leishmaniasis have various inadequacies including drug resistance and a lack of effective control and eradication of the disease spread. Therefore, the development of a rationale-driven, target based approaches towards novel therapeutics against leishmaniasis is an emergent need. The utilization of Artificial Intelligence/Machine Learning methods, which have made significant advances in drug discovery applications, would benefit the discovery process. In this review, following a summary of the disease epidemiology and available therapies, we consider three important leishmanial metabolic pathways that can be attractive targets for a structure-based drug discovery approach towards the development of novel anti-leishmanials. The folate biosynthesis pathway is critical, as *Leishmania* is auxotrophic for folates that are essential in many metabolic pathways. *Leishmania* can not synthesize purines *de novo*, and salvage them from the host, making the purine salvage pathway an attractive target for novel therapeutics. *Leishmania* also possesses an organelle glycosome, evolutionarily related to peroxisomes of higher eukaryotes, which is essential for the survival of the parasite. Research towards therapeutics is underway against enzymes from the first two pathways, while the third is as yet unexplored.

## 1. Background

**Figure 1:**
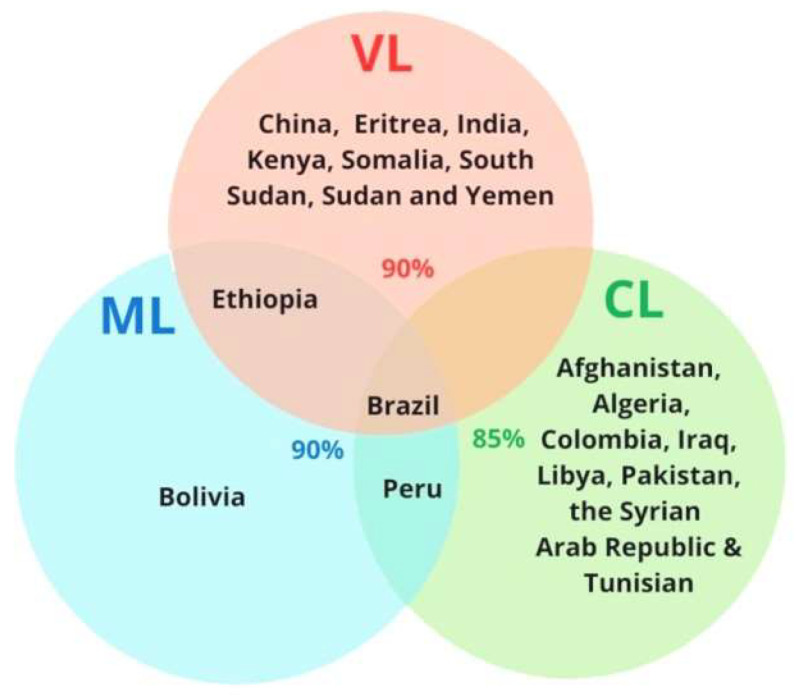
**Global incidences of three different types of leishmaniasis.** Venn diagram shows the countries affected by different types of leishmaniasis (WHO 2020) [created with BioRender.com accessed on [4 April 2022]].

Leishmaniasis is caused by the protozoan parasites consisting of ~20 *Leishmania* species, transmitted through the bites of promastigote-infected female phlebotomine sandflies during a blood meal. In the mammalian host, promastigotes are engulfed by phagocytic cells, transform into amastigotes within the cells, rapidly multiply and become a new source of infection back to the vector through the next blood meal [1]. The three main forms of the diseases have distinct symptoms and differ in degrees of severity. Visceral leishmaniasis (VL), also known as kala-azar (in the Indian subcontinent) [2], is the most lethal form of the disease, resulting in fatality if untreated. The clinical symptoms and signs of VL include irregular bouts of fever, weight loss, enlargement of the spleen and liver, and anemia; cutaneous leishmaniasis (CL), the most common form of leishmaniasis, mainly causes skin lesions and induces ulcers, which could leave life-long scars with serious disabilities; mucocutaneous leishmaniasis (ML) leads to the partial or total destruction of tissues in the nose, mouth, and throat [3]. The symptoms of leishmaniasis thus vary, ranging in pathogenesis from moderate in CL to extreme in VL. The disease affects many countries worldwide with Algeria, Afghanistan, Bolivia, Brazil, China, Colombia, Eritrea, Ethiopia, India, Iraq, Kenya, Libya, Pakistan, Somalia, South Sudan, Sudan, Syrian Arab Republic, Tunisia, and Yemen accounting for ~90% of the infections (Figure 1) [3].

**Figure 2:**
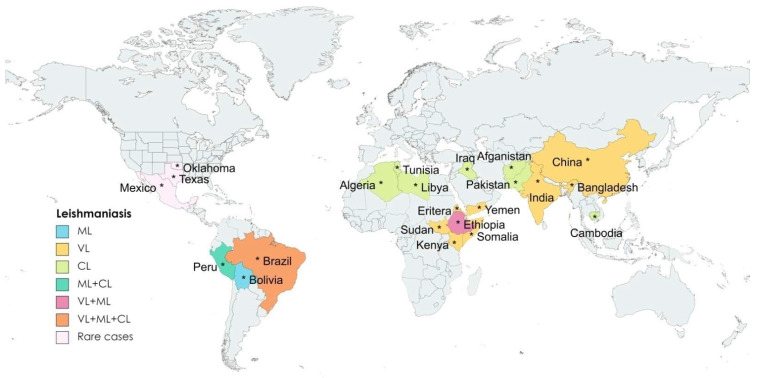
**Distribution of various leishmaniasis in different countries:** Mapping the global prevalence of three types of leishmaniasis reported across the globe (* are used to indicated the correct location of countries on the map) (adapted from the World Health Organization, 2020) [Created with MapChart.net].

Leishmaniasis has also been reported in some regions of Mexico, Central America, and South America, and some cases of cutaneous leishmaniasis have been noticed in Texas and Oklahoma in the USA (Figure 2) [3]. The epidemiology of leishmaniasis depends on the characteristics of the parasite and sand fly species, the local ecological features of the infection sites, the history of parasitic infection in the human population, and human physiology [3]. 

Although several drugs are available for chemotherapy of leishmaniasis, almost all of them have significant drawbacks (Table 1) [4]. Most anti-leishmanial treatments require multiple doses, for a prolonged period of time, contributing not only to the difficulty in the management of clinical leishmaniasis but also to the development of drug resistance. (Table 1) [5,6].

Most anti-leishmanial drug therapies are cumbersome and not always effective [20] and vaccines are still in the developmental stage. Alternative therapies such as the application of cytokines and immune modulators have also been investigated together with chemotherapy [21,22,23]. Significantly, these alternative approaches are also in the preliminary stage and do not ensure protection against the disease [24]. The developing vaccines are mostly prophylactic and can be broadly classified into three categories: live attenuated *Leishmania* vaccines e.g., ascorbic acid-deficient live mutants of *Leishmania donovani*; dead parasite vaccine (*Leishmania major* (ALM) + BCG vaccine); or defined vaccines such as DNA vaccines gP63 for visceral leishmaniasis. It is thus imperative that intensive focused research on rationale-driven candidates and target-based drug discovery be initiated towards obtaining novel therapeutics against leishmaniasis, utilizing approaches including structure-based drug design (SBDD) and ligand-based drug design (LBDD) approaches. 

**Figure 3:**
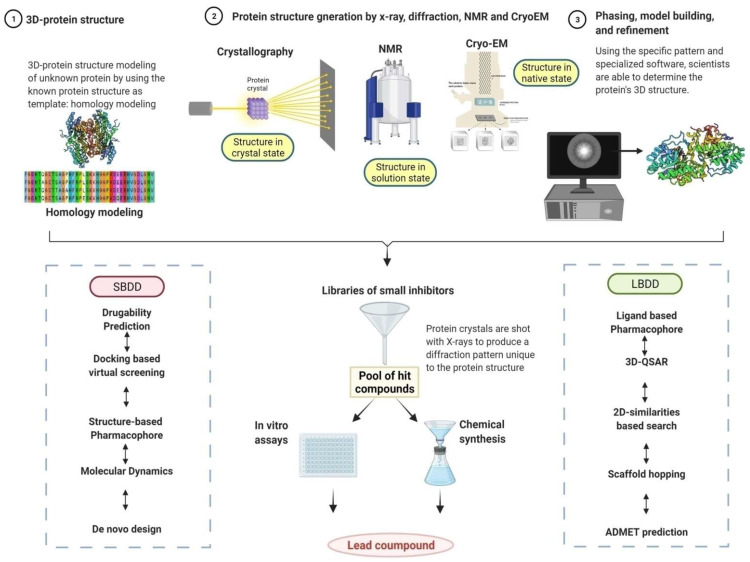
**Workflow for target-specific drug discovery and its utilities in the *Leishmania*-specific inhibitor discoveries.** Drug discovery approaches begin with the determination of 3D structures of target protein either by X-ray crystallography, NMR, cryo-EM or by using computational methods (homology modeling). This serves as the template for molecular docking studies against a library of chemical entities, resulting in the identification of lead molecules, i.e., specific hit compounds based on the binding score and binding dynamics. Identified molecules would be experimentally bio-assayed and in parallel facile chemical methods would be established. The working process of SBDD starts with druggability prediction, followed by docking-based virtual screening and pharmacophore identification. Finally, molecular dynamics would provide insights into the protein followed by lead optimization. If the structure of the protein is unknown, the ligand-based drug discovery (LBDD) approach would be utilized to design an analog molecule after refinement by scaffold hopping. The main aim of scaffold hopping is the discovery of any novel chemical moiety with improved pharmacological features compared with the marketed drug against the same target protein. It starts with ligand-based pharmacophore identification, followed by structure–activity relationship analysis. Finally, the drug-discovery system deals with the various properties such as chemical absorption, distribution, metabolism, excretion, and toxicity (ADMET) of any lead compound (future drug) [created with BioRender.com].

In structure- and ligand-based drug-discovery approaches, two components, small chemical inhibitors and target proteins and enzymes, are the central players. The major modules of SBDD comprise (i) docking the chemical inhibitors from chemical inhibitor libraries to the identified target proteins followed by (ii) experimental investigations to understand the effects of the selected docked inhibitors on the enzymatic activities in a multi well or high-throughput format. In the absence of a target protein 3D structure, structure–activity relationships (SARs)-based series of analogs could be synthesized and rescreened for the generation of potent lead compounds against the target protein. The LBDD strategy is used when SBDD is not possible due to the unavailability of the target protein structure for the fast and cost-efficient discovery of lead molecules (Figure 3).

The first step in an SBDD is the identification and preparation of the target protein’s three-dimensional structure (experimentally elucidated or computationally modelled) for *in silico* screening (Figure 3), followed by the docking of libraries of small molecules to the target protein using appropriate software and web-based tools and ranking. The interaction between protein and candidate molecules predicted via docking in SBDD provides details of the orientation and surface complementation of the interacting atoms. After the ligand is docked to the target protein, SBDD effectively refines the protein-ligand structure to improve ligand binding within the protein active site, in a process defined as lead-optimization. Additionally, molecular dynamics simulations may also be performed to understand and optimize the relative orientations of the interacting atoms to achieve a more favorable configuration (Figure 3). On the other hand, LBDD is used predominantly when the structure of the target protein is unknown (or cannot be modelled) but the effects of the chemical modulator on the target protein activity are known. LBDD primarily employs structure-activity relationships (SARs), where several analogs of the natural modulator would be designed with similar structure and chemical properties. If many modulators are known for the target protein, utilizing machine learning (ML) algorithms could be helpful for virtual screening in obtaining potent analogs. Scaffold hopping, which is extensively used in medicinal chemistry to generate several iso-functional compounds with distinct core moieties, could also be utilized in LBDD. The final step of any drug discovery (common to SBDD and LBDD) is to understand the pharmacokinetics and pharmacodynamics of the lead compounds which includes absorption, distribution, metabolism, excretion, and toxicity (ADMET) parameters (Figure 3) [25,26,27,28].

**Figure 4:**
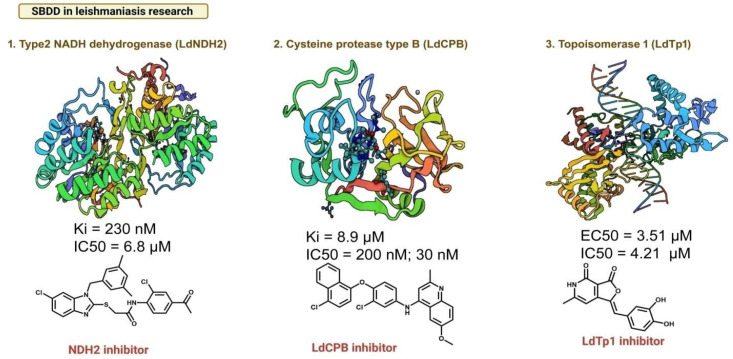
**SBDD-based inhibitors discoveries.** Selected examples are showing the inhibitor discoveries against various enzymes by employing SBDD approaches under the umbrella of anti-leishmanial research [created with BioRender.com].

SBDD approaches have been successful in the identification of a novel inhibitor compound and lead molecules against a variety of diseases including infectious diseases, with some of them reaching clinical trials and getting FDA approvals. These approaches to the discovery of therapeutics against NTDs including the development of novel anti -leishmanials have made remarkable progress [28,29,30]. SBDD approaches using the structures of leishmanial Cysteine Protease B (CPB; essential for parasite survival in the host), Type 2 NADH dehydrogenase [NDH2] (a mitochondrial enzyme that converts NADH to ubiquinone and is crucial for parasite survival), Nucleoside Diphosphate Kinase (crucial for maintaining intracellular NTP levels), and Topoisomerase-II (catalyzes single-stranded breaks in DNA during gene replication and transcription) have led to the identification of novel inhibitors (Figure 4) [28,31,32,33,34,35,36]. It is pertinent, however, to emphasize that these studies have not yet resulted in the development of candidates that have reached any clinical trial stage but are primary steps towards a rationale-driven approach for novel anti-leishmanials. As *Leishmania* diverged early, significant differences are observed between leishmanial and mammalian homologs, whether in the individual proteins or in the whole pathways. For example, the Adenosine methyl decarboxylase (S-AdometDC) involved in the synthesis of polyamines, is an essential enzyme for all cellular organisms including *Leishmania*. Surprisingly, kinetoplastids including *Leishmania* have an additional enzyme, the AdometDC-like protein (ADL), which forms a hetero-oligomer with AdometDC, substantially increasing the efficacy. *Leishmania donovani* ADL also appears to bind to substrates while other trypanosomatid homologs have not been reported to bind to any substrates [37,38], indicative of subtle differences within close evolutionary related homologs as well. A similar distinction among close homologs is also observed in the C-terminal Coiled-coil (CC) oligomerization domain of the actin-interacting protein coronin that plays a role in actin-dynamics. Structurally, the *Leishmania donovani* and *Trypanosoma brucei* CC domains have a novel topology, oligomeric association, and an asymmetry, compared with other coronin CC structures, though the magnitudes of the asymmetry are different [39,40].

## 2. Emergence of Artificial Intelligence (AI) in Anti-leishmanial Drug Discovery

Artificial intelligence (AI) is a machine learning algorithm used to predict, analyze, interpret, model, and decide the further steps to handle very complex and bulky information in an efficient, fast, and reliable manner [41]. AI in medicine has modified the working model of health services from appointment scheduling, translating clinical data, and diagnosis, to decreasing healthcare costs [42,43,44]. Moreover, in the era of omics research, the emergence of AI would help in the analysis of high volumes of data and also provide virtual assistance during the entire research program [45,46,47,48]. Though AI enhances the therapeutic discovery processes significantly, an overemphasis on AI is also not recommended, as its drawbacks include susceptibility to security risks, inaccuracies in calculations being subsequently carried over, overlooking social variables, and human surveillance is still a necessity [49]. In drug-discovery research, AI contributes to quantitative structure-activity and property relationship (QSAR), structure-based modeling, *de novo* molecular design, prediction of physiochemical properties such as solubility, and partition coefficient of drug and chemical synthesis prediction [50,51,52]. Methodologically, AI works through machine learning (ML) mode by employing deep learning (DL) which finally constructs artificial neural networks (ANNs) [53] employing various algorithms, models, and approaches such as random forest, k-nearest neighbors (KNN), support vector machines (SVM), multilayer perceptron (MLP), learning vector quantization (LVQ), multi pass LVQ, linear discriminant analysis (LDA), artificial neural networks (ANN), Viola–Jones algorithm, and gradient boosting approaches [54].One of the best examples of an AI system is ‘AlphaFold Protein Structure Database’ which has been developed by DeepMind and EMBL-EBIand models the 3D structure from the amino acid sequences. This database covers the complete human proteome and 47 other species including *Leishmania* [55]. AI in anti-leishmanial drug discovery is still an emerging field and at an early phase that certainly requires extensive exploration at various levels such as predicting the protein structure, studying the interaction with inhibitors, projecting the favorable structure–activity relationship, mining the possible pharmacological features, suggesting the facile chemical synthesis processes, modeling the future cost analysis for actual drug discoveries, prediction of clinical trial failure, listing the adverse effects of the future drug, suggesting post-treatment prognosis situations, and advising of alternative medical treatments. AI in leishmaniasis research has contributed to critical aspects including infection diagnosis from microscopic images, peptide-fingerprints-based prediction and modeling of enzyme classes, pyruvate kinase inhibitors designing, prognosis features of unresponsive patients, and alternative chemotherapy prediction [56,57,58,59,60].

## 3. Parasite-Specific Essential Metabolic Pathways and Possible Drug Targets

Rationale-driven novel therapeutic approaches focus primarily on proteins and enzymes of metabolic pathways. As *Leishmania* is an eukaryote as are humans, the chosen pathway has to be either absent in humans or significantly diverged (sequence, tertiary structure, active site architecture and composition) for effective therapeutics. A recent review details various biochemical pathways which are crucial for the *Leishmania* parasite [61]. The rest of this review, for brevity, focuses on three key leishmanial metabolic pathways which could be plausible contenders for future anti-leishmanials: the folate biosynthesis pathway which is also parasite specific; the purine salvage pathway, as *Leishmania* cannot synthesize purines *de novo* and are dependent on the host for their purine requirements; and the peroxisomal import pathway. While the purine salvage and folate biosynthesis pathways have received considerable attention in relation to the development of anti-leishmanials, the peroxisomal import machinery is as yet unexplored.

### 3.1. Folate Biosynthesis Pathway

**Figure 5:**
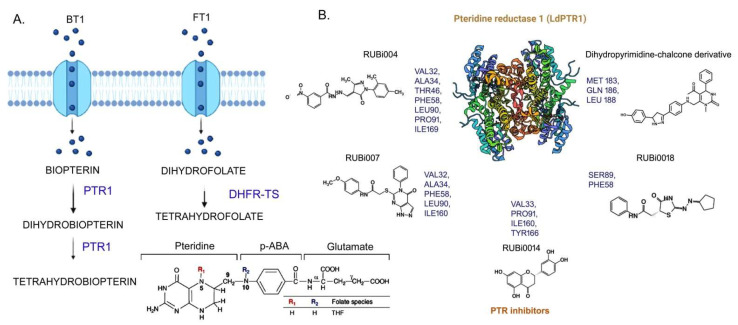
**Folate biosynthesis pathway in *Leishmania donovani* and targeting of LdPTR1 enzyme with specific inhibitors.** (**A**)—BT-1 (biopterin) and FT-1 (folate) enter in the parasitic cells through transporters. Thereafter, metabolic enzymes such asPTR1 and DHFR-TS catalyze further steps and produce tetrahydrobiopterin and tetrahydrofolate, respectively. (**B**)—PTR-1 protein interacts with various inhibitors through the indicated amino acids, primarily involving H-bonds and π-π interactions (created with BioRender.com).

Folic acid plays an essential role in key metabolic inter conversions in all forms of life which involve or result in the transfer of C1 (one carbon) units [62]. Folates, alternate carbon sources that donate or accept one carbon in many metabolic pathways, are heterocyclic derivatives of pterins and pteroic acid (conjugated with p-aminobenzoic acid pABA and glutamates (Figure 5A), that act as co-factors in hydroxylation, oxidoreduction, and C1 transfer reactions, and also have a significant role in many biological processes including DNA replication and amino acid metabolism [63,64].Surprisingly, while prokaryotes (and certain lower eukaryotes) can synthesize folate and folic acid *de novo* as they have catalytic enzymes for folate biosynthesis [62,65,66,67], higher eukaryotes including mammals lack these enzymes and pathways and are dependent on dietary resources via membrane-associated transport [62,65,66,67].

The multistep folate synthesis starts with the GTP cyclohydrolase I (GTPCHI) catalyzed pterin ring synthesis from GTP as a precursor, mainly in plants and prokaryotes [42,43]. In the fourth and penultimate step, dihydropteroate synthase mediates pABA attachment with pterin and after the completion of the reaction cycle, each catalyzed by a specific enzyme, GTP is converted into 7,8-dihydrofolate, reduced by dihydrofolate reductase (DHFR) to produce 5,6,7,8-tetrahydrofolate (folate or vitamin B9) [62,65,66,67]. In contrast, pterin ring synthesis in higher eukaryotes (including humans) occurs in three steps, starting with the precursor GTP, via the formation of 7,8-dihydroneopterin triphosphate (H2NTP), 6-pyruvoyl-tetrahydropterin (PTP), and finally into tetrahydrobiopterin (BH4) catalyzed by GTP cyclohydrolase I (in humans coded by the gene GCH1), 6-pyruvoyl-tetrahydropterin synthase (coded by PTS), and sepiapterin reductase (coded by SPR) enzymes, respectively [62,65,66,67]. Historically, the activity of biopterin as a cofactor for amino acid hydroxylases in any organism was identified by its requirement for the growth of the trypanosomatid insect trypanosome, *Crithidia fasciculate* [68,69,70,71,72]. Prokaryotes and many eukaryotes can synthesize both unconjugated and conjugated pteridines, but kinetoplastid protozoans require exogenous pteridines for their survival [73]. *Leishmania* acquire folates by active transport, using two different transporters that were identified by functional studies of mutants resistant to methotrexate (MTX) [71]. 

The three transporter and catalytic enzymes of folate biosynthesis in *Leishmania,* summarized in Table 2 are: (i) pterin reductase, (ii) thymidylate synthase (TS), and (iii) dihydrofolate reductase (DHFR) (Figure 5A) [73]. Gene deletion studies show that deletion of either PTR1 or DHFR-TS is lethal for the viability of *Leishmania*, especially in the intracellular environment of the host where folate and thymidilate rescue routes are insufficient. Additionally, PTR1 knockout *Leishmania* was hypersensitive to MTX and could not survive in a medium lacking pterins [74]. Thus, a dual inhibition approach of PTR1 and DHFR-TS would be promising for the development of potential drugs for the treatment of leishmaniasis.

Pterinreductase (PTR1) is a conserved dehydrogenase enzyme, with its catalytic site formed by Tyr, Lys, Ser, and Asn residues [68,69,73]. However, the serine in the leishmanial PTR1 catalytic site is replaced by an aspartate. To date, there are 14 PTR1 experimental structures reported for *Leishmania*: (12 from *Leishmania major* and one each of *Leishmania tarentolae* and *Leishmania donovani*) [77]. SBDD approaches with leishmanial PTR1 as the target protein have led to the discovery of novel inhibitors that combine the features of dihydropyrimidine and chalcone derivatives against PTR1 with improved efficacies (Figure 5B).

The other two main enzymes related to folate metabolism, dihydro folate reductase (DHFR), and thymidine synthase (TS) are commonly found fused as a bifunctional enzyme in some protozoa including *Leishmania*, unlike the monomeric enzymes found in bacteria and mammals [78,79,80,81]. Experimental structures of DHFR-TS from *Leishmania* are still unknown, although preliminary crystallographic data of the *Leishmania major* DHFR-TS complex have been reported, while structures of *Trypanosoma* homologs that share a (65–70%) sequence identity are available [78,79,80,81]. The human DHFR has ~35% sequence identity. The structures indicate that the bifunctional enzyme has the N-terminal DHFR domain and the C-terminal TS domain, with the linker region significantly shorter (three amino acids, compared to the long linkers in apicomplexa) [78,79,80,81]. The active site of the human enzyme is comparatively smaller and less hydrophobic, suggesting that more lipophilic inhibitors could potentially form stronger interactions with the protozoan enzymes. Such findings might precisely guide the discovery of new potential inhibitors against DHFR with a highly lethal capacity for parasites. Recently, research works have reported the development of molecules with activity against PTR1 and DHFR-TS of *Leishmania*, utilizing cheminformatics features to determine the binding mode and the possible activity in such enzymes [78,79,80,81,82,83,84].

### 3.2. The Purine Salvage Pathway

Unlike their mammalian and insect hosts, *Leishmania* completely lack *de novo* nucleotide biosynthesis pathways, are entirely dependent on their salvage pathway from hosts to fulfill their purine requirement, and are considered as obligate purine auxotrophs [85]. The *Leishmania* purine salvage pathway has three major components: extracellular nucleotidases (ecto-nucleotidase) that hydrolyze extracellular nucleotides into nucleosides to maintain the extracellular nucleoside pool, and also finally facilitate their transportation inside the parasite; transporter systems responsible for transporting purines through the cellular membrane; and catalytic enzymes for further processing of the salvaged purines, as required (Table 3) [86].

#### 3.2.1. Extracellular Nucleotide Metabolism in *Leishmania* by Ecto-Nucleotidase

**Figure 6:**
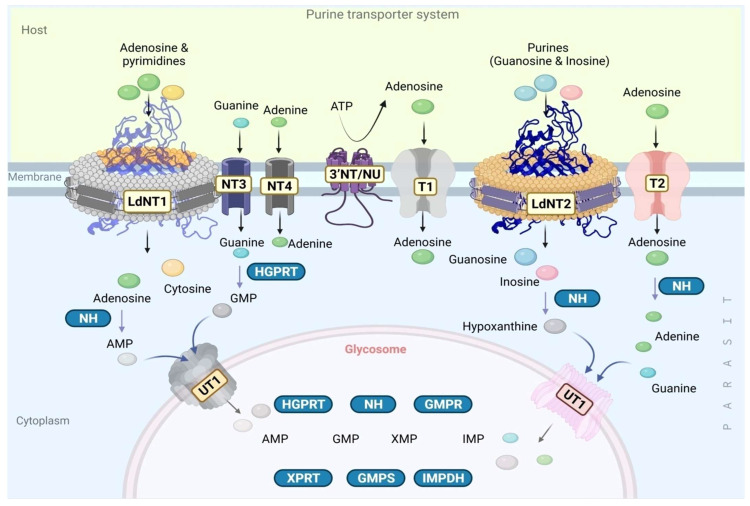
**Purine transporter system in *Leishmania.*** Ecto-nucleotidases (*Ld*3’NT/NU) hydrolyze the nucleotides (ATP) into nucleosides (adenosine). Adenosine and purine transportation occurs through LdNT1/2, NT3, NT4, T1, and T2 transporters across the cell membrane from the host. Various enzymes such as HGPRT of the purine salvage pathway (shown in blue boxes) catalyze the conversion of guanine to guanine mono phosphate. GMP, AMP hypoxanthine, adenosine, and guanine transportations into glycosome mediated by unknown transporters 1 and 2 (UT1/2) [created with BioRender.com].

The nucleobases-specific transporter proteins in *Leishmania* usually require nucleosides instead of nucleotides. The pool of nucleosides is maintained by parasite-specific membrane-bound ecto-nucleotidases, enzymes which hydrolyze nucleotides into nucleosides (Figure 6) [87]. It has been shown that the most virulent *Leishmania* species (*Leishmania amazonensis*) efficiently hydrolyzes higher amounts of ATP, ADP, and AMP (Figure 6) [87]. Nucleotide hydrolysis by parasitic ecto-nucleotidases enzymes (membrane-bound) can modulate the host’s immune response effectively which finally results in the establishment of infection. Parasitic (including leishmanial) ecto-nucleotidases mediated hydrolysis of ATP and nucleotides disrupt the ‘danger signal’ (marked by the accumulation of excessive ATP and pro inflammatory cytokines at the site of infection) for many immune cells and prohibit the activation of any Th2 or pro inflammatory host immune responses [88]. Ecto-5’ nucleotidase (known as CD73 invertebrates) in *Leishmania* could also hydrolyze the ATP to supply the nucleosides [88]. Interestingly, *Leishmania donovani* possesses an exclusive ecto-3’ nucleotidase (*Ld*3’NT/NU) that could hydrolyze both nucleotides and nucleic acids including RNA and ssDNA [89,90] that are projected as putative drug targets [86]. 

#### 3.2.2. The Purine Transporter Systems

*Leishmania* parasites are decorated with specific transporters for the uptake of nucleobases primarily on the plasma and glycosomal membranes (Figure 6) [91,92,93,94,95,96,97,98]. Nucleobase transporters (NTs) NT1 and NT2, are permease proteins that exclusively transport inosine and guanosine. In addition, two supportive transporters NT3 and NT4 assist the transportation of nucleobases across the parasite membrane from the host. Moreover, it has been shown that in infectious amastigotes, two additional transporters T1 and T2 also carry adenosines with high affinity [85]. After import into the cytoplasm, the cytoplasmic salvage pathway enzymes including nucleoside amino hydrolases (NH) [for conversion to AMP, hypoxanthine, and adenine], HGPRT convert guanine into GMP [91]. Subsequently, AMP, hypoxanthine, and GMP enter the glycosome through an unknown transporter -1 (UT1) [91], which can directly transport adenine and guanine to the glycosome without any cytoplasmic enzymatic reactions. Interestingly, these transporters are pH-dependent (both neutral and acidic) and could switch the kind of nucleosides for transportation, which facilitate their survival even inside the acidic environment of phagolysosome [91]. Finally, glycosome-specific salvage pathway enzymes use these precursors or intermediates and catalyze the production of nucleotides (nucleoside mono-, di-, and triphosphates), fulfilling the parasite’s requirements (Figure 6) [91,92,93,94,95,96,97,98]. Comparative sequence studies of *Leishmania* and human nucleoside transporters reveal that 16 amino acids are conserved [96]. These conserved amino acids are hypothesized to be important for structure, based on structural analyses with other permeases. *Ab initio* computational modeling studies indicate that *Ld*NT1, which comprises 11 transmembrane domains, undergoes a significant movement during its function with the transmembrane helices adopting inward-open and outward-close conformations [99], stabilized by two additional aromatic residues Phe 48 (TM1) and Trp 75 (TM2). Significantly, changes in the non-conserved amino acids could alter the functioning of NTs, as evinced by the point mutations G183D (TM5) and C337Y (TM7), which reduced the adenosine transportation by ~20 fold [99,100], opening a new window for precise structural and functional investigations of each amino acid in relation to drug discovery [99,100]. Structure-based identification of small inhibitors which could either block, freeze, or interlock the confirmation of these transporters or switch the substrate selectivity (or kinetics) and hinder purine transportation might be a novel idea to target *Ld*NT1. These hypotheses await the elucidation of NT structures for further validation.

#### 3.2.3. Purine Salvage Enzymes

**Figure 7:**
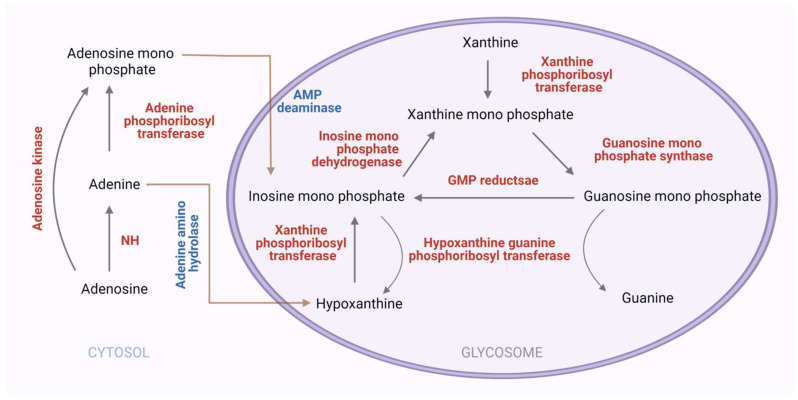
**The purine salvage pathway which is catalyzed by specific enzymes in *Leishmania* (predicted pathway).** The predicted purine salvage pathway in *Leishmania*, the pink compartment represents glycosomes. Enzymatic reactions catalyzed by indicated proteins of the purine salvage pathway in *Leishmania* [created with BioRender.com].

The major enzymes of the purine salvage pathway in *Leishmania* are Phosphoribosyltranferases (PRTs) which convert dephosphorylated purines into corresponding nucleoside monophosphates [91]. *Leishmania* expresses three different PRTs: adenine-phosphoribosyltransferase (APRT) (EC2.4.2.7); hypoxanthine-guanine phosphoribosyltransferase (HGPRT) (EC2.4.2.8); and xanthine phosphoribosyltransferase (XPRT) (EC 2.4.2.22). In *Leishmania*, two distinct routes are found for adenine salvage via adenine phosphoribosyltransferase (APRT) or adenine amino hydrolase (AAH), converting adenine to hypoxanthine and AMP, respectively (Figure 7). Genetic deletion studies of APRT and AAH individually and in combination, confirm the importance of these enzymes with AAH playing a dominant role in purine salvage, with the hypoxanthine subsequently salvaged by HGPRT or XPRT [91]. The majority of purine flux into nucleotides occurs via two main routes, either HGPRT or XPRT, despite superficial complexity and convolutedness. HGPRT catalyzes the conversion of hypoxanthine to inosine monophosphate (IMP) and guanine to guanosine monophosphate (GMP). The crystal structures of *Leishmania donovani* APRT in complex with the substrate adenine and product AMP and sulfate and citrate ions that appear to mimic the binding of phosphate moieties, reveal differences in the binding poses with the active site pocket in a more open conformation to accommodate the larger AMP ligand [101]. Whereas AMP adopts a single conformation, adenine binds in two mutually exclusive orientations: one an orientation providing adenine-specific hydrogen bonds orientation and the other postulated for positioning adenine for the enzymatic reaction [101]. The structure of *Leishmania donovani* HGPRT, when compared with the human homologs (PDB ID: 1HMP), reveals contrasting modes of oligomer association involving distinct regions of the proteins, which could be valuable in rational drug discovery approaches [102]. XPRT acts on two substrates, XMP and diphosphates, forming xanthine and 5-phospho-alpha-D-ribose1-diphosphate. Among all PRT enzymes of the purine salvage pathway in *Leishmania*, XPRT (xanthine phosphoribosyltransferase) is unique in its substrate specificity and its nonexistence in humans, so it is an interesting protein not only for drug designing but also to understand the molecular determinants of its substrate specificity. A computational model of *Leishmania donovani* XPRT hypothesized that residues Ile 209, Tyr 208, and Glu 215 are important for the altered substrate specificity [102,103]. The enzymes of the purine salvage pathway are attractive therapeutics in *Leishmania* parasite, though the structures for a majority of these enzymes are yet unknown.

### 3.3. Peroxisomal Import Pathway

Peroxisomes are organelles that are found in almost all higher organisms from yeast to humans. Peroxisomes are surrounded by a single phospholipid bilayer membrane. The peroxisomal matrix harbors various enzymes that are involved in various biochemical processes including beta-oxidation, gluconeogenesis, and the pentose pathway. In yeast and plants, beta-oxidation of fatty acid takes exclusively in the peroxisome, while in mammals, the beta-oxidation of long-chain fatty acid takes place in the peroxisomes and shorter chain beta-oxidation occurs in mitochondria [104]. In plants, certain steps of photorespiration also take place in peroxisomes [104]. Dysfunction of peroxisomal proteins leads to various diseases in humans such as the Zellweger syndrome [105,106,107,108], neonatal adrenoleukodystrophy (NALD) [109], and infantile Refsum disease [110,111]. Kinetoplastids including *Leishmania* and *Trypanosma* possess the organelle glycosomes that are primarily derived from the peroxisome [112]. Glycosomes contain enzymes related to glucose metabolism and other related pathways such as the purine salvage pathway [113], and PEX proteins play a major role in the biogenesis of peroxisome. To date, around 34 peroxins are known of which functions of 19 are directly linked with peroxisome biogenesis [114,115,116]. It has been shown that during peroxisome biogenesis, the peroxisome either transports the peroxisomal protein into its membrane or destines the protein to the ER which eventually would make the membrane of the peroxisome [114,115,116]. Characterization of proteins of this pathway in *Leishmania* (or other kinetoplastids) are in infancy and consequently, there are no inhibitors identified against any of them. Table 4 summarizes the structural and inhibitor details of proteins of the leishmanial peroxisome import pathway.

**Figure 8:**
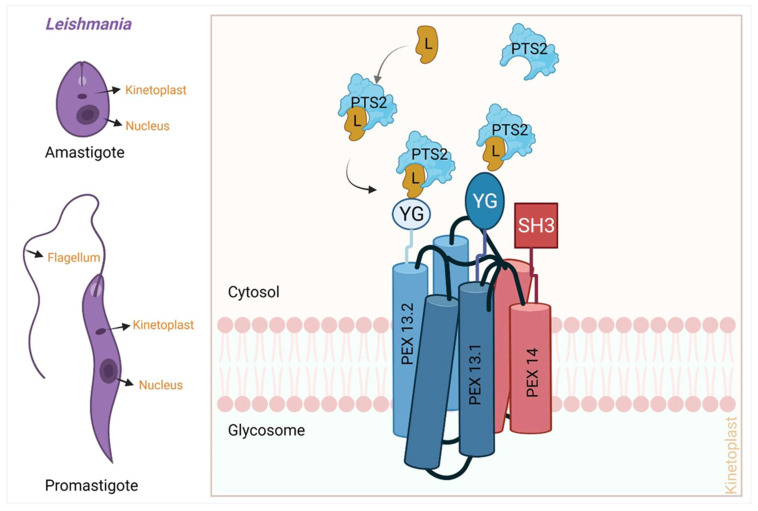
**Peroxisomal import receptors (PEX), mediated import of cytoplasmic protein into peroxisome of *Leishmania* at amastigote and promastigote stages.** Peroxisomal transport sequence (PTS) containing cargo proteins first bind with the Ligand protein, which facilitates the delivery of these cargos to the PEX receptors. Once these cargo proteins are loaded over to the PEX complex then they selectively control their import inside the peroxisome [created with BioRender.com].

The peroxisome import can be divided into the following stages (Figure 8):

(a) Recognition of cargo in the cytosol
(b) Loading of cargo or receptor complex onto the peroxisomal membrane
(c) Cargo translocation over the membrane
(d) Release of cargo complex into the peroxisomal membrane
(e) Receptor recycling

Newly synthesized peroxisomal matrix proteins are transported to their desired location with the help of targeting signal sequences known as a peroxisomal targeting sequence (PTS). Two types of PTS are found, a conserved C-terminal PTS-1 (peroxisomal target sequence 1) and the N-terminal nona peptide PTS-2 (peroxisomal target sequence-2). The presence of the PTSs in any protein sequence does not always imply their peroxisomal targeting. Upon synthesis of glycosomal enzymes in the cytosol, they are recognized by two cytosolic receptors, PEX5 and PEX7. PEX5 recognizes PTS1 through its C-terminal tetra tricopeptide repeat (TPR), while PEX7 recognizes PTS-2. As PEX5 also contains a PEX7 binding region, the ultimate import depends on PEX5.The cargo-loaded receptors dock at the glycosomal membrane by binding to PEX14. PEX5-PEX14 binding results in the formation of a dynamic transient import pore, which allows translocation of the enzymes into the glycosomal lumen [112,117,118,119]. The peroxisomal import mechanism of *Leishmania* (Figure 8) involves the cytoplasmic PTS containing proteins binding with specific cargo protein [120]. Briefly, the peroxisomal transport signal-containing proteins which are initially present in the cytoplasm first bind with the specific ligand protein (L). This cargo-loaded ligand (L) protein has an affinity to bind PTS-proteins and facilitates the cargo delivery at the PEX receptors. Then PEX13 and 14 control the import of these cargo proteins into the peroxisome [119]. The glycosomal membrane-associated *Leishmania donovani* protein PEX14 plays a crucial role in protein import from the cytosol to the glycosomal matrix and consists of three domains: an N-terminal domain where the signaling molecule binds, a trans membrane domain, and an 84-residue coiled-coil domain (CC) that is responsible for oligomerization [120]. As seen in Table 4, no structures for any of the components of this important pathway from *Leishmania* are available, though a few trypanosomatid homologs are known [120]. Recently, a crystallization note of the CC domain of *Ld*PEX14 has appeared in the literature [121], and any structural deviations and its critical functional values could be a great opportunity for SBDD [31,104]. The potential value of this pathway for a therapeutic approach thus depends on the structural elucidation of the components.

## 4. Conclusions

Leishmaniasis, a seriously debilitating NTD is still a major issue due to limited disease management, with high global morbidity. Current drugs such liposomal amphotericin B, paromomycin, and oral miltefosine have contained the disease to a certain extent, but have associated inadequacies including drug resistance, necessitating immediate discovery of novel anti leishmanials. As *Leishmania* majorly depends on the human host for their basic metabolic requirements, it has adapted several unique features and protein machinery which ultimately ensure their survival even under the adverse environment of the human host cells. Research is constantly underway to delineate suitable drug targets and is mainly focused on the protein related to important metabolic pathways, precursor transportation through glycosome, and adaptation machinery. Here, we have discussed three metabolic pathways and listed crucial enzymes, which are putative targets for therapeutic approaches. Some of these proteins have already been characterized in SBDD and novel inhibitors identified with potential activity inhibition and cytotoxicity (Figure 3). Recently, extensive work has been performed to filter the crucial drug targets by applying SBDD, LBDD, and other approaches, and has successfully discovered putative inhibitors. However, in vitro and in vivo validations need further exploration as they could provide an effective targeted future anti-leishmanial drug with minimal side effects. Structural characterization of leishmanial proteins precludes a rationale driven approach, despite the whole genome sequencing of *Leishmania* being completed in 2005 [122]. *Leishmania major* has 36 chromosomes with a haploid genome size of the 32.8 megabase, consisting of 911 predicted RNA genes, 39 pseudogenes, and 8272 protein-coding genes. In contrast, a query word search of ‘*Leishmania*’ in the Protein Data Bank (PDB) (in July 2022), resulted in only 632 entries. The corresponding numbers are higher for *Trypanosoma* where ~1378 structures are reported. These numbers reflect that the major obstacle in the application of SBDD in *Leishmania* is the unavailability of tertiary or quaternary structures of many leishmanial proteins, which might play critical roles in various metabolic pathways for housekeeping functions and survival. However, the computational prediction of protein structures using artificial intelligence and machine learning approaches as in AlphaFold2 and RoseTTA fold enable SBDD approaches to be carried forward in the absence of experimental structural data [123]. The robust and cohesive approach involving the characterization of the ~8000 leishmanial proteins on fast-track mode would pave the way for the development of novel anti-leishmanials, and also provide insights into parasite biology and their evolution. Advancements in the AI and machine learning areas also complement the anti-leishmanial drug discovery field and minimize human error with a more focused strategy.

## Figures and Tables

**Table 1:** Current chemotherapy for visceral leishmaniasis.

Drug	Discovery	Mode of Action	Side Effect	Remark
**Pentavalent antimonial** [7]	Brahmachari, 1940 [8]	Proposed: Trypanothione Reductase and macromolecule biosynthesis inhibitor	Cardiotoxicity, injection pains	Development of drug resistance
**Amphotericin B (1992)** [9]	Repurposed antifungals for neutropenic patients [10].	Cell membrane permeabilization via a complex with sterols and ergosterols	Renal toxicity, hypokalemia	Dose-limiting
**Liposomal amphotericinB (2009)** [11,12]	Used in antifungal infections, e.g., *Cryptococcal meningitis* [13]	Targeted drug delivery to infected macrophages to kill amastigotes therein	Minor fever, chills, arthralgia, and rarely renal toxicity	Expensive cold-chain storage required
**Miltefosine (2002)** [14]	Repurposed anticancer drug 1980s [15].	Modulate cell surface receptors and inositol metabolism, apoptotic cell death, cytochrome C oxidase inhibitor	Teratogenicity, GI, and hepato–renal toxicity	Expensive
**Paromomycin (1960)** [16]	Used to treat certain intestinal parasites [17]	An aminoglycoside binds to 30S ribosomal subunit, inhibiting protein biosynthesis, decreasing membrane potential	Nephro- and ototoxicity, reversible high tone audiometric shift	Expensive
**Pentamidine (1940)** [18]	Repurposed from anti trypanosomiasis [19]	Mitochondrial topoisomerase II and transcription inhibitor	Gastrointestinal disorder, hypotension, diabetes mellitus	Expensive

**Table 2:** *Leishmania* folate biosynthetic enzymes: sequence similarities with human homologs and available inhibitors.

Protein or Enzyme	Comparative Analysis of *Leishmania* Andhuman Homologs			Specific Inhibitors: Discovery and Approach	
	Sequence Identity (%) *Leishmania* vs. Human	Structural Classification ^@^		*Leishmania*	Human
		*Leishmania*	Human		
DHFR-TS (Dihydro folate reductase-thymidylate synthase) *L. donovani, L. infantum, L. amazonensis, L. major*	Dihydro folate reductase (60–61.2%) Thymidylate synthase (60–61%)	N/A	Reductase ^a^ Transferase ^b^	Structure-based prediction only: Withaferin-A (withanolide) which binds to the active sites of human DHFR and human TS, but binds to a site other than an active site in *L. donovani* DHFR-TS [75]	Activity based-Withaferin-A discovery against DHFR and TS enzymes
PTR1 (Pteridine reductase) *L. major*	L-xylulosereductase (36.8%)	Oxidoreductase ^c^	N/A	1-Structure-based: benzimidazole/benzoxazole derivatives [61] 2-Structure-based: RUBi004, RUBi007, RUBi014, RUBi018 [76]	N/A
PTR1 (Pteridine reductase) *L. donovani*	Dehydrogenase and reductase SDR family member 4 -like 2 (48.8%)	Oxidoreductase ^d^	N/A	N/A	N/A
PTR1 (Pteridine reductase) *L. tarentole*	3-oxoacyl-[acyl-carrier-protein] reductase (37.7%)	Oxidoreductase ^e^	N/A	N/A	N/A

^@^ The structural Classification is as annotated in the corresponding Protein Data Bank (PDB) entries of the leishmanial and human homologs. The PDB IDs (unique 4-letter alphanumeric codes, starting with a numeric value) of the individual entries correspond to: ^a^—1DHF, ^b^—1HZW, ^c^—2QHX, ^d^—2XOX, ^e^—1P33 while N/A indicates no structural details are available, to the best of our knowledge.

**Table 3:** *Leishmania* purine salvage pathway enzymes: sequence similarities with human homologs and available inhibitors.

Protein or Enzyme	Comparative Analysis from *Leishmania* and Human Homologs			Specific Inhibitors: Discovery and Approach	
	Sequence Identity (%) *Leishmania* vs. Human	Structural Classification ^@^		*Leishmania*	Humans
		*Leishmania*	Human		
NT4 (Nucleobasetransporter 4)	Nucleobase transporter 4 (30.2%)	N/A	N/A	N/A	N/A
APRT (Adenine phosphor ribosyltransferase)	Adenine phosphor-ribosyltransferase (35.12%)	Transferase ^a^	Transferase ^b^	N/A	Structure-based: iminoaltritolbis-phosphates (L-DIAB and D-DIAB) in *Plasmodium falciparum* [26]
HGPRT (Hypoxanthine phosphoribosyltransferase)	Hypoxanthine-guanine phosphoribosyltransferase (34%)	Transferase ^c^	Transferase ^d^	N/A	Structure-based: Immucillin GP in humans [27]
XPRT (Xanthine phosphoribosyltransferase)	Xanthine phosphoribosyltransferase (33.8%)	N/A (only modeled with hHGPRT)	N/A	Structure-based: Ld-XPRT inhibitors (dGDP and cGMP) [28]	N/A
AAH (Adenine aminohydrolase)	Adenosine deaminase (23.3%)	N/A	Aminohydrolase ^e^	N/A	N/A
GDA (Guanine deaminase)	Guanine deaminase (34.9%)	N/A	Aminohydrolase ^f^	Enzyme kinetics-based: N6-methyladenine (6-methylaminopurine [6-MA] [29]	N/A
GMPS (Guanosine mono phosphate synthase)	GMP synthase (48.1%)	N/A	Ligase ^g^	N/A	N/A
(AK) Adenosine Kinase	Adenosine Kinase (36.7–41.4%)	N/A	Kinase ^h^	N/A	N/A
ADSS (Adenylosuccinate Synthetase)	Adenylosuccinate Synthetase (28.5–32.4%)	Ligase ^i^	Ligase ^j^	N/A	N/A
ASL (Adenylosuccinate Lyase)	Adenylosuccinate Lyase (25.3%)	Lyase ^k^	N/A	N/A	N/A
AMPDA (Adenosine mono Phosphate deaminase)	AMP deaminase (47.5%)	N/A	Hydrolase ^l^	N/A	N/A

^@^ The structural Classification is as annotated in the corresponding Protein Data Bank (PDB) entries of the leishmanial and human homologs. The PDB IDs (unique 4-letter alphanumeric codes, starting with a numeric value) of the individual entries correspond to: ^a^ 1QCD, ^b^ 1ORE, ^c^ 7CMJ, ^d^ 1Z7G,. Ids that start with prefix—AF are the predicted protein structures by AI based database (AlphaFold). ^e^ 3IAR, ^e^ 7RTG, ^e^ AF-P00813-F1, ^f^ 2UZ9, ^f^ 3E0L, ^f^ 4AQL, ^f^ AF-Q9Y2T3-F1, ^g^ 2VPI, ^g^ 2VXO, ^g^ AF-P49915F1, ^h^ 1BX4, ^h^ 2I6A, ^h^ 2I6B, ^h^ 4O1L, ^h^ AF-P55263-F1, ^i^ AF-A7LBL2-F1, ^j^ 2V40, ^j^ AF-P30520-F1, ^k^ AF-Q01432-F, ^l^ 4MX2, while N/A indicates no structural details are available, to the best of our knowledge.

**Table 4:** *Leishmania* peroxisomal import pathway enzymes: Sequence similarities with human homologs and available inhibitors.

Protein and Enzyme	Comparative Analysis of *Leishmania and* Human Homologs			Specific Inhibitors: Discovery and Approach	
	Sequence Identity (%) *Leishmania* vs. Human	Structural Classification ^@^		*Leishmania*	Human
		*Leishmania*	Human		
PEX3 (Peroxisomal targeting signal-1 receptor 3) [114,115,116]	A-kinase anchor protein 9 (24.1%)	N/A	N/A	N/A	N/A
PEX5 (Peroxisomal biogenesis factor 5) [114,115,116]	Peroxisomal targeting signal 1 receptor (26–31%)	N/A	Peroxins ^a^	N/A	N/A
PEX11 (Peroxisomal biogenesis factor 11) [114,115,116]	Probable UDP-sugar transporter protein SLC35A5 (34.1%)	N/A	N/A	N/A	N/A
PEX13 (Peroxisomal biogenesis factor 13) [114,115,116]	Peroxisomal membrane protein PEX13 (43.7%)	N/A	Peroxins ^b^	N/A	N/A
PEX14 (Peroxisomal membrane protein) PEX14 [114,115,116]	Peroxisomal membrane protein PEX14 (43.1%)	N/A	Peroxins ^c^	N/A	N/A
PEX-7 (Peroxisomal biogenesis factor 7) [114,115,116]	Peroxisomal biogenesis factor 7 PEX7 (30.21%)	N/A	N/A	N/A	N/A

^@^ The structural Classification is as annotated in the corresponding Protein Data Bank (PDB) entries of the leishmanial and human homologs. The PDB IDs (unique 4-letter alphanumeric codes, starting with a numeric value) whereas Ids that start with prefix—AF are the predicted protein structures by AI based database (AlphaFold): ^a^ 2C0L, ^a^ 2C0M, ^a^ 2J9Q, ^a^ 2W84, ^a^ 3R9A, ^a^ 4BXU, ^a^ 4KXK, ^a^ 4KYO, ^b^ AF-Q92968-F1, ^c^ 2W84, ^c^ 2W85, ^c^ 4BXU while N/A indicates no structural details are available, to the best of our knowledge.

## Data Availability

Not applicable.

## Abbreviations

Neglected tropical disease (NTD), Visceral Leishmaniasis (VL), Cutaneous Leishmaniasis (CL), Mucocutaneous Leishmaniasis (ML), Structure-based drug design (SBDD), Ligand-Based Drug Design (LBDD), Pseudo-structure–activity relationship (PSAR), Quantitative Structure–activity Relationship (QSAR), Pteridine reductase 1 (PTR1), Cysteine proteases type B enzymes (CPB), Type 2 NADH dehydrogenase (NDH2), Topoisomerase 1 (Top1), Nucleobase transporter (NT), Unknown transporter (UT), Transmembrane domain (TM), Phospho ribosyltranferase (PRT), Adenosine mono phosphate (AMP), Guanosine mono phosphate (GMP), Xanthosine mono phosphate (XMP), Adenine phospho ribosyltransferase (APRT), Hypoxanthine-guanine phosphoribosyltransferase (HGPRT), Xanthine phosphoribosyltransferase (XPRT), Adenosine kinase (AK), Adenine aminohydrolase (AAH), Guanine deaminase (GDA), Adenylo succinate synthetase (ADSS), Adenylo succinate lyase (ASL), Adenine Mono Phosphate deaminase (AMPDA), Inosine monophosphate dehydrogenase (IMPDH), Guanosine mono phosphate synthase (GMPS), Guanosine mono phosphate reductase (GMPR), Nucleoside hydrolase (NH), Adenosine (ADO), Adenine (ADE), Coiled coil (CC).
